# What Are Shared Emotions (for)?

**DOI:** 10.3389/fpsyg.2016.00412

**Published:** 2016-03-24

**Authors:** John Michael

**Affiliations:** Department of Cognitive Science, Central European UniversityBudapest, Hungary

**Keywords:** shared emotions, minimalism, joint action, development, phenomenology, coordination, cooperation

In recent years, a burgeoning literature has been developing around the topic of shared emotions, addressing such questions as whether there is any such thing as a shared emotion, and what functions shared emotions may have. A quick survey of that literature reveals that different researchers have proposed to conceptualize shared emotions in different ways—and that at least some participants to the debate take these different proposals to be incompatible.

In this brief discussion, I will be arguing that these different proposals are in fact not incompatible, and that they only appear to be incompatible if one assumes that there is just one natural kind for which the term “shared emotion” should be reserved. In the absence of any compelling argument in favor of adopting this assumption, I suggest that it should be dropped, and that we should acknowledge that the term “shared emotion” refers to a motley of overlapping phenomena that do not make up a single natural kind. In view of this, the most productive way forward is for researchers to be as clear as possible about what they are trying to explain in proposing a particular conception of shared emotions. To begin with, consider four different conceptions that have recently been proposed.

The first is one that I myself presented with the aim of demarcating a range of phenomena that are systematically related to each other and that can function as coordinating factors in joint actions, and to specify the conditions under which they can so function (Michael, 2011). To this end, I proposed the following minimal criteria. For two individuals, X and Y, there is a shared emotion when:

- (a) X expresses her affective state (verbally or otherwise); and
- (b) Y perceives this expression;

In view of the aim of specifying the conditions under which shared emotions can function as coordinating factors in joint actions, I also proposed to limit discussion to shared emotions that also fulfill a third criterion, namely that:

(c) Y's perception of X's expression leads to effects that function as coordinating factors within an interaction between x and Y.

Shared emotions in this sense can facilitate coordination by facilitating the exchange of information between X and Y about how X evaluates the current situation. For example, X's expression of frustration may indicate that she needs additional support or that the plan is not working and needs to be revised.

Now consider a second conception of shared emotions, which at first glance appears quite different from the minimal account. Salmela and Nagatsu (2016), propose that X and Y have a shared emotion when X and Y:

(d) have emotions of the same type with similar intentional structure and affective experience; and
(e) are mutually aware of this.

Salmela and Nagatsu, like myself, are guided by the aim of explaining how shared emotions can serve particular functions within joint actions. They wind up with a different set of criteria because the particular functions they are interested in are different. Specifically, they are interested in how shared emotions can generate social cohesion, trigger team reasoning and stabilize cooperation. Thus, the differences between the two accounts can be traced back to their related but distinct explanatory aims: Salmela and Nagatsu's account is designed to explain how shared emotions support individuals' *motivation* to engage in joint action and to contribute to each other's goals and shared goals, whereas mine was mainly designed to show how shared emotions support individuals' *ability* to coordinate.

A third conception of shared emotions can be found in Krueger (2013; cf. also Krueger, 2014). Krueger argues that the concept of shared emotions is a useful tool for explaining how infants come to experience some emotions. Specifically, he points out that emotional experience places demands upon endogenous attention and affective regulation that exceed the capacities of infants, and that caregivers scaffold infants' emotional experience by fulfilling these functions for (and together with) children in dyadic interactions. Insofar as these endogenous attention and affective regulation are typically part of an emotional episode, parity of reasoning demands that the contributions of caregivers to these emotional episodes should also be considered part of the emotional episodes (for criticism, see Bohl and Mölder, 2015). Plainly, Krueger's conception of shared emotions is quite different from the previous two: rather than stipulating any particular criteria for shared emotions, he argues that the criteria for emotional episodes in general sometimes are fulfilled by dyads rather than individuals.

Finally, Zahavi and Rochat (2015) propose a conception of shared emotions which differs markedly from both of the preceding proposals. On their view, shared emotions do not require x and y to have emotions of the same type [i.e., they reject conditions (d) and (e)]. Instead, they propose that “emotional sharing involves experiences that the subjects can only have in virtue of their reciprocal relation to each other, experiences that rather than being independent of each other are constitutively interdependent. Shared emotional sharing is consequently something over and above empathy^1^. It adds reciprocity and co-regulation to the understanding that is provided by empathy” (Zahavi and Rochat, 2015, p.7).

It is apparent that the conception offered by Zahavi and Rochat differs from the other three conceptions. Indeed, Zahavi and Rochat appear to believe that their conception is incompatible with at least two of the aforementioned conceptions: contra Salmela and Nagatsu^2^, they maintain that sharing, “cannot simply be equated with similarity,” (p.6) They go on: “Nor is it plausible to argue, as Michael has done in a recent article, that emotion detection amounts to a minimal form of sharing and that a paradigmatic way of sharing an emotion is for X to express an affective state, and for Y to perceive that expression…To claim that I am (aware of) sharing one of your emotions, while denying that you are (aware of) sharing one of mine, does not seem to make that much sense.”

Although Zahavi and Rochat do not explicitly state why they dismiss these other views, they appear to be reasoning that there is a particular type of experience which their conception captures and which the other conceptions do not capture. If so, then there is a problem: Zahavi and Rochat need to indicate what basis they have for asserting that the expression “shared emotion” should be reserved for this type of experience. In the absence of any reason for adopting the assumption that shared emotions constitute a natural kind, it is open to proponents of alternative conceptions to acknowledge happily that Zahavi and Rochat provide an analysis of their target phenomenon, but to reply that they are interested in some other explananda.

In view of the diversity of conceptions of shared emotions that are available in the current literature, it is justified to conclude that the expression “shared emotion” is in fact used to refer to a motley of phenomena that do not make up a single natural kind. This is not a problem: different conceptions of shared emotions are suited to different explanatory purposes, and should accordingly be tailored to those explanatory purposes. In the interest of constructive debate, then, it would be highly desirable for researchers to be as clear as possible about what they are trying to explain in proposing a particular conception of sharing emotions.

This does not mean that the different explanatory targets, or the different conceptions of shared emotions that are tailored to those targets, are likely to have nothing at all to do with each other. Indeed, at least some of them may relate to each in systematic ways. In fact, one way of thinking about the relationship between my own account and that offered by Salmela and Nagatsu is that both accounts start out from criteria (a) and (b)^3^ and then add different further criteria according to their different explanatory aims. If this is correct, then it shows how a minimal framework like the one presented in Michael (2011) may provide a useful tool for relating at least some of these different accounts. This is because a minimal framework starts out from as few controversial assumptions or criteria as possible. Depending on what one wants to explain, one may add assumptions and criteria accordingly^4^. This is also a productive research strategy since it makes it possible to identify what additional conditions are required in order to explain what specific phenomena.

## Author contributions

The author confirms being the sole contributor of this work and approved it for publication.

### Conflict of interest statement

The author declares that the research was conducted in the absence of any commercial or financial relationships that could be construed as a potential conflict of interest.

## Footnotes

^1^For the sake of clarity, it is worth pointing out that, in referring to empathy, Zahavi and Rochat have in mind the traditional phenomenological concept of empathy, where empathy “rather than being identified with, say, prosocial behavior or a very special kind of imaginative perspective taking, is simply used as a label for our most basic other-acquaintance, i.e., our sensitivity to and direct experience of other minded creatures” (Zahavi and Rochat, 2015, p.15). In other words, empathy, as they understand it, does not require an empathizer to experience a same emotion of the same type as the person with whom she is empathizing, and indeed it does not require either person to experience any emotion at all. “rather than being identified with, say, prosocial behavior or a very special kind of imaginative perspective taking, is simply used as a label for our most basic other-acquaintance, i.e., our sensitivity to and direct experience of other minded creatures” (Zahavi and Rochat, 2015, p.15). In other words, empathy, as they understand it, does not require an empathizer to experience a same emotion of the same type as the person with whom she is empathizing, and indeed it does not require either person to experience any emotion at all.

^2^NB: They are not specifically discussing Salmela and Nagatsu's proposal here.

^3^Salmela and Nagatsu do not explicitly endorse these criteria, but, as far as I can tell, the fulfillment of these criteria is likely to be the most basic and most common way in which criterion (e) is fulfilled.

^4^This is also the logic underlying minimal approaches in other areas, such as the minimal approach to joint action offered in Vesper et al. (2010) and the minimal approach to the sense of commitment offered in Michael et al. (2016).
